# Partial Recovery in Toxic Leukoencephalopathy: Is It Really a Slow Improvement or a Warning Sign?

**DOI:** 10.7759/cureus.42966

**Published:** 2023-08-04

**Authors:** Jigar P Mankad, Kyle Paulsen, Mihir Shah

**Affiliations:** 1 Neurology, Aurora St. Luke's Medical Center, Milwaukee, USA; 2 Neuro Critical Care, Aurora St. Luke's Medical Center, Milwaukee, USA

**Keywords:** toxic encephalopathy, chasing the dragon encephalitis, delayed post hypoxic encephalitis, cocaine induced encephalitis, encephalitis

## Abstract

A 55-year-old African American man who was found down by a friend nine hours after being last seen at the same place was brought to the emergency department (ED) with encephalopathy, lactic acidosis, rhabdomyolysis, elevated troponin, acute kidney injury (AKI), and transaminitis. His urine drug screen (UDS) was positive for cocaine. Intravenous (IV) Narcan was given with minimal improvement in mental status. A computed tomography (CT) scan of the head and a CT scan of the cervical spine in the ED showed no acute findings. Due to hypoxia, the patient was eventually intubated. The patient also required a fasciotomy and eventually hyperbaric oxygen (HBO) therapy due to the left lower extremity wound. He was transferred to our facility for further care. Due to incomplete cognitive recovery, as the patient was oriented to self only, further neurological workup, including magnetic resonance imaging (MRI) of the brain, was obtained, which showed bilateral symmetric T2 FLAIR (Fluid attenuated inversion recovery) hyperintensity in the globus pallidus. The patient had slow and gradual deterioration with worsening encephalopathy, akinetic mutism, parkinsonian features, and seizures, which prompted further evaluation from neurology. The patient eventually underwent extensive workup, including a continuous video electroencephalogram (cvEEG), repeat MRI brain with and without contrast, lumbar puncture for cerebrospinal fluid (CSF) analysis, MRI brain with diffusion tensor imaging (DTI), and magnetic resonance spectroscopy (MRS). The patient was treated with multivitamin therapy and coenzyme Q10, but there was no significant benefit. We report a case of cocaine-induced leukoencephalopathy with findings like ‘chasing the dragon encephalopathy’ with a possible component of delayed post-hypoxic injury with underlying neuroinflammation.

## Introduction

Altered mental status is one of the most difficult diagnoses or consults any provider can decipher. The list can range from infectious to structural pathology to ictal secondary to seizures. Leukoencephalopathy is any disorder that affects the white matter or the axonal fiber tracts [1]. Toxic leukoencephalopathy is an even more specific subset of white matter diseases that can be associated with exogenous substances such as alcohol abuse (Wernicke’s Encephalopathy, Marchiafava-Bignami disease), industrial agents (methanol, toluene), inhaled gases (carbon monoxide, pesticides), chemotherapeutic agents (methotrexate, fludarabine, 5-fluorouracil), immunosuppressive agents (tumor necrotic factor (TNF)-α blockers, cyclosporine), other potentially neurotoxic medications (metronidazole, vigabatrin), or illicit drugs (heroin, cocaine) [1,2]. Toxic leukoencephalopathies pose a significant risk of catastrophic brain damage if not diagnosed earlier [2]. Pursuing a structured approach and choosing the correct imaging modality is important to reach the diagnosis systemically, from the most common to the least common etiology [2]. Heroin is the most common opioid used in the intravenous or inhaled form, and it is very well reported in the literature due to classic MRI of the brain findings, commonly known as the ‘chasing the dragon’ form [2]. Patients with suspected intravenous or inhaled drug intoxication are at high risk of toxic leukoencephalopathy with incomplete recovery, neurobehavioral symptoms, abnormal movements, extrapyramidal rigidity, parkinsonian features, or progressive neurological deterioration [3-5]. Clinicians require a high index of suspicion and should consider detailed drug screens, including synthetic opioid screens, as cases of benzodiazepine as well as non-opioid substances can also cause toxic leukoencephalopathy [6,7].

## Case presentation

**Figure 1:**
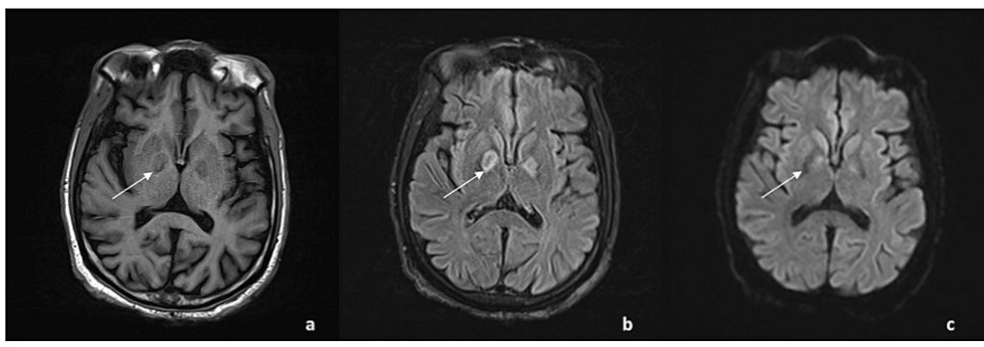
Initial MRI brain imaging Bilateral symmetric globus pallidus signal abnormality: (a) hypointense on T1 (b) hyperintense on FLAIR,  and (c) absence of diffusion restriction on DWI

A 55-year-old African American man with a known history of bipolar disorder, glaucoma, and cocaine use was brought to the emergency room by EMS after a friend found the patient unresponsive after seeing him at the same place nine hours before EMS’s (Emergency Medical Services) arrival. The patient had minimal cognitive recovery with the initial Narcan given by EMS and a repeat dose in the emergency department. The patient was hemodynamically stable but found to have lactic acidosis, rhabdomyolysis, elevated troponin, acute kidney injury (AKI), and transaminitis. His urine toxicology screen was positive for cocaine, and no opioids were found. Per the friend, the patient was snorting cocaine and a possible unknown substance. The serum drug screen and carbon monoxide screening were negative. Initial computed tomography (CT) of the head, the facial bones, and the cervical spine showed no acute findings. Due to multiorgan involvement and hypoxia, he was eventually intubated. The patient was placed on continuous venovenous hemofiltration (CVVH). Due to prolonged pressure on the left leg, the patient developed compartment syndrome and required a fasciotomy. Finally, the patient was extubated one week after the hospitalization and transferred to the floor. The patient was more alert and able to communicate, but cognitive recovery remained incomplete. He was reported to be calm and appropriate but confused and disoriented to time and place and oriented to self. No other focal findings on the cranial nerve exam or focal weakness in the extremities existed. The patient was transferred to our hospital 13 days after the initial presentation for hyperbaric oxygen therapy for the left lower extremity wound. As the patient was disoriented to time and place questions, had a nasogastric tube for dysphagia, and was not cleared by speech for swallowing, a magnetic resonance imaging (MRI) brain without contrast was requested to rule out a possible global hypoxic injury or posterior circulation stroke. However, the likely etiology of his encephalopathy was toxic metabolic in origin. His brain MRI showed symmetric FLAIR hyperintensity in the bilateral globus pallidus with no diffusion restriction; these findings were concerning for possible carbon monoxide poisoning versus opioid-induced encephalopathy (Figure 1).

**Figure 2:**
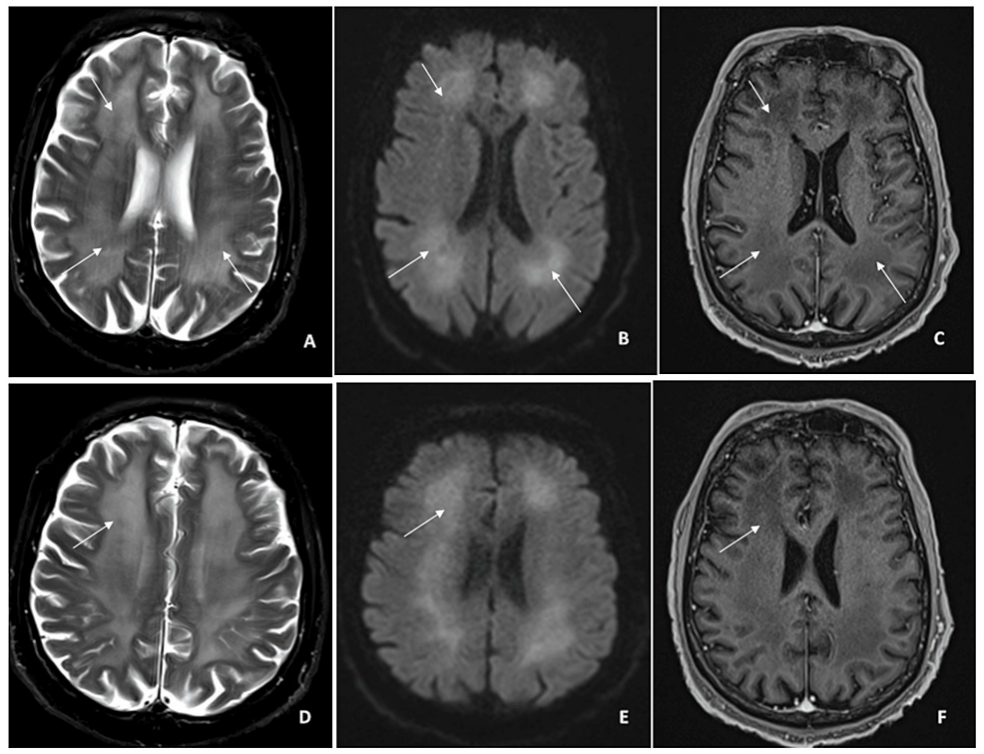
Follow-up MRI brain imaging Interval development of extensive and diffuse cerebral white matter (A and D) T2-FLAIR hyperintensity and (B and E) diffusion restriction, symmetrically involving all the lobar white matter without cortical involvement. Hypointense (C and F) T1 images

The patient endorsed using illicit drugs but could not name the substances; he remained disoriented when asked time and place questions. There was ongoing concern about his left lower extremity wound and uremia contributing to his mental status changes, and the patient continued to get CVVH. He remained oriented toward himself, and eventually, neurology was consulted due to a lack of cognitive recovery. During the initial evaluation, the patient was stuporous, disoriented to time and place, had no evident cranial nerve deficit, and had increased tone in bilateral upper and bilateral lower extremities, resulting in bilateral hip and knee flexion posture with difficulty relaxing or lying flat, withdrawal from painful stimulation, and no focal weakness in extremities. Psychiatry was also consulted for evaluation 20 days after his hospitalization, as the patient was less communicative and avoided eye contact. He was diagnosed with major depressive disorder and polysubstance use-related mood disorder, and the patient's family also mentioned that the patient used cocaine frequently. Between days 21 and 28, the patient slowly became less verbal, delirious, tremulous, unable to participate in the therapy well, and more dependent on transfer out of bed. Due to the findings of hypoactive delirium and catatonia on the exam, psychiatry was requested to re-evaluate and consider a possible trial of benzodiazepines. A routine electroencephalogram (EEG) was ordered, which did not show any focal slowing or epileptiform discharges or diffuse slowing suggestive of possible diffuse cortical dysfunction or toxic metabolic or hypoxemic encephalopathy. The patient was eventually started on a lorazepam trial for catatonic features, low-dose carbidopa-levodopa due to concern of drug-induced parkinsonism causing extrapyramidal rigidity and treated with high-dose thiamine empirically. Due to his body posture, an MRI brain was very difficult. The patient eventually had a generalized tonic-clonic seizure associated with tachypnea and tachycardia, followed by a prolonged postictal state; he was transferred to the neuro-intensive care unit and intubated for airway protection. The patient was monitored for 24 hours on continuous video EEG, confirming no further seizures or ongoing status epilepticus. MRI brain was obtained after the patient was intubated, which revealed diffuse cerebral white matter T2 FLAIR hyperintensity and diffusion restriction, as well as hypointense T1 signal bilaterally, along with clinically progressive encephalopathy over four weeks, most compatible with progressive toxic metabolic injury versus opiate-induced leukoencephalopathy (Figure 2).

**Figure 3:**
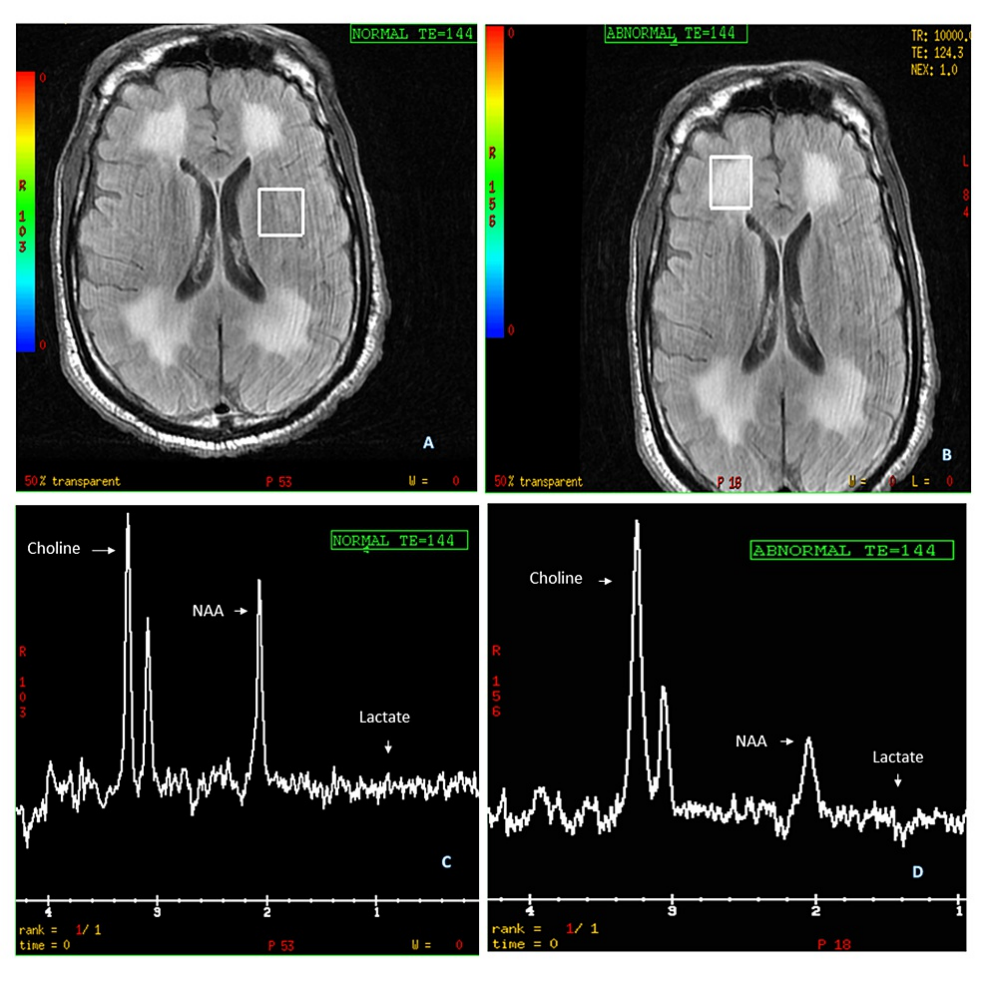
MR spectroscopy MR spectroscopy shows an elevated choline peak in (A) normal as well as (B) abnormal regions of interest and a decreased NAA peak in (C) normal and (D) abnormal regions of interest. Absent lactate peak.

His encephalopathy was presumed to be related to possible opioid-induced use (likely heroin use) based on the MRI brain features, although his initial drug screen was positive only for cocaine. The patient was empirically started on treatment with coenzyme Q10, vitamin E, and vitamin C. He was also started on levetiracetam (Keppra) for seizure prevention. Due to the uncertainty of substance ingestion before hospitalization, possible differential diagnoses of acute disseminated encephalomyelitis, progressive multifocal leukoencephalopathy, Wilson's disease, atypical posterior reversible encephalopathy syndrome, systemic lupus erythematosus, and carbon monoxide poisoning were also considered. The patient underwent extensive evaluation with cerebrospinal fluid (CSF) analysis for rapid meningitis/encephalitis panel, JC (John Cunningham) virus panel, multiple sclerosis screening, Mayo Clinic paraneoplastic panel, Mayo Clinic autoimmune encephalitis panel, and glycine receptor antibody, which eventually came back negative. The patient initially had elevated CSF proteins suggestive of central nervous system (CNS) inflammation. Still, subsequent lumbar punctures showed normalization of CSF protein and no elevated WBC count in the CSF at any time. Serum workup for JC virus antibody was positive, suggesting previous exposure, but negative for CSF polymerase chain reaction (PCR) for JC virus. His serum workup for an antinuclear antibody with a reflex panel, thyroid, and anti-cytoplasmic nuclear antibodies also returned negative. The patient was also screened for immunoglobulin levels to rule out any underlying immune deficiency. A brain biopsy was also considered, but neurosurgery suggested completing a workup. Eventually, the family refused the brain biopsy. The patient eventually underwent an MRI brain with diffusion tensor imaging (DTI) and MRI brain spectroscopy, which showed an elevated choline peak in normal as well as abnormal regions, a decreased NAA (N-Acetyl aspartate) peak in normal and abnormal areas of interest, an absent lactate peak, findings of diffuse supratentorial white matter changes, and evolving injuries within the globus pallidus and right caudate nucleus most compatible with toxic metabolic damage causing inflammation and neuronal edema (Figure 3).

Synthetic opioid screening was also carried out, but it was probably very late and turned out negative. It could not be added to the previous blood collection from the initial hospitalization. The patient eventually required a tracheostomy and (percutaneous endoscopic gastrostomy) PEG tube placement; he was alert but not interactive, had increased tone in all four extremities, and responded to tactile stimulation in all four extremities. He did not have significant cognitive recovery at the time of discharge to a long-term acute care facility.

## Discussion

Drug-induced toxic leukoencephalopathy can be seen with prescription medications like metronidazole or vigabatrin and is less commonly associated with heroin, cocaine, and benzodiazepine overdoses in rare circumstances [2,8]. Cocaine is known to cause multiple neurological adverse conditions, including cerebral ischemia, posterior reversible encephalopathy, vasospasm, and cerebral edema [9]. Among the rare causes of toxic leukoencephalopathy, which leads to delayed post-hypoxic encephalopathy several days or weeks after the initial presentation, are those commonly associated with opioid use [7,10]. The potential cause of delayed hypoxic-ischemic encephalopathy is thought to be secondary to widely spaced linear arterioles and a lack of anastomosis amongst the deep white matter, making it vulnerable to hypoxic injuries [11]. A few reports of heroin-induced spongiform leukoencephalopathy and further animal studies showed induction of cerebellar granular cell apoptosis and c-Jun N-terminal kinase (JNK) pathway activation as the primary underlying mechanisms. JNK inhibitor trial medications were also used in animals to suppress toxic effects induced by heroin; nothing similar has been found for cocaine-induced leukoencephalopathy [12,13]. These illicit drugs have been shown to induce apoptosis and cell death by activating the inflammatory cascade. Immunohistochemical analyses have also demonstrated collapse and fracture of the myelin sheath and eventual vacuole formation, which leads to spongiform encephalopathy, commonly affecting areas of the brain like the subcortical white matter, cerebellum, corpus callosum, and deep gray matter nuclei [14]. 

Subacute progressive neurological decline with the initial finding of leukoencephalopathy on MRI brain is commonly associated with heroin vapor inhalation, also known as chasing the dragon encephalopathy. Typically, patients progressively worsen after the initial presentation [4,5,15]. Rare cases have been related to the sole use of benzodiazepine, which led to toxic leukoencephalopathy [7]. Several cases have been reported lately where patients had initial recovery from severe encephalopathy or a comatose state after opioid overdose, followed by the delayed syndrome of sudden progressive neurological deterioration, which may include behavioral and cognitive changes, confusion, extrapyramidal stiffness, akinetic mutism, catatonic features, automatism, seizure-like activity, and gait difficulties, which may progress to quadriparesis, stupor, or even coma [7,16]. Cocaine-induced leukoencephalopathy may have a different trajectory of symptoms characterized by disorganized behavior, increased muscle tone, catatonic features with rhabdomyolysis, and rarely hyperthermia, which is likely induced by excessive dopaminergic dysregulation and which may be a physiological variant of the neuroleptic malignant syndrome [17]. This is somewhat different in patients with heroin-induced leukoencephalopathy, where patients have an initial phase with more cerebellar symptoms followed by extrapyramidal rigidity and more spasm and akinetic mutism later in the course [17]. Cocaine is more commonly associated with cerebrovascular events, and cocaine-related leukoencephalopathy is rare, possibly related to some underlying autoimmune etiology and possible underlying neuroinflammation by substances typically mixed with cocaine, like levamisole [18]. 

Illicit drug use or acute intoxication can cause altered mental status, and the patient can present with encephalopathy. Many patients respond to Narcan effectively, but a patient may have a partial or poor response. Some patients may have progressive deterioration, leading to requirements for respiratory support and intensive care unit admission. Frequent neurochecks are essential to continuously assess the mental status of these patients to assess for improvement or deterioration in their clinical condition. Continuous video EEG in intensive care unit patients is essential in 24-48 hours to rule out underlying nonconvulsive status epilepticus. Patients with persistently altered mental status beyond the initial 48 hours require a structured, thorough approach to identify the underlying etiology. Most of the patients have a CT scan of the head completed in the emergency department. An early MRI of the brain is helpful to assess for any drug-induced ischemic or hypoxic process or ruling out any ischemia secondary to vasospasm. Based on the areas involved in the brain with white matter, changes can also help to suggest the underlying diagnosis. Patients with partial improvement in mental status or poor cognitive recovery should undergo further evaluation, including synthetic drug screening, lumbar puncture for cerebrospinal fluid analysis, rule out any underlying central nervous system infection, and considering possible underlying paraneoplastic or autoimmune encephalitis pathology [8]. When the MRI brain suggests diffuse leukoencephalopathy, initial or synthetic drug screens can help identify likely offending agents. MRI findings of diffuse periventricular white matter involvement with sparing basal ganglia and cerebellum involvement can raise suspicion for likely underlying opioid-induced leukoencephalopathy. Deep gray matter nuclei involvement can be seen with intravenous as well as inhaled heroin vapor and rarely with cocaine use [2,17-19]. Magnetic resonance imaging may show multiple tumefactive T2 FLAIR hyperintense lesions that mimic an underlying demyelinating disorder, with or without gadolinium enhancement. Patients may sometimes receive treatment with intravenous steroids due to suspicion of an underlying demyelinating disease [18]. MR spectroscopy can further help to assess the possible route of drug intoxication; inhaled heroin vapor commonly causes an elevation of the lactate peak compared to intravenous heroin or cocaine inhalation, which can show an elevated choline peak in normal as well as abnormal areas of the brain [20]. Patients with diffuse leukoencephalopathy, likely related to toxic etiologies like acute drug intoxication, have been treated with high doses of vitamin C, E, and coenzyme Q. However, it lacks evidence to support any benefit. Due to NMDA receptor antagonism, some patients have been treated with memantine, considering its role in treatment [9]. Patients were also treated empirically with high-dose steroids during the early phase. However, there was no substantial evidence to support its use, which may enhance recovery in these patients. In some cases, partial recovery has been reported with the help of steroids, plasmapheresis, or immunoglobulin, but the overall neurological outcome can be unfavorable. The overall mortality rate of up to 20%-30% has been reported in different cases [18].

## Conclusions

Our paper describes an interesting case of toxic cocaine-induced leukoencephalopathy with incomplete cognitive recovery initially followed by delayed worsening of symptoms, concerning possible opioid-induced pathology with an absence of opioids on the initial drug screen. Cocaine-induced leukoencephalopathy cases have been reported in the past but are not very common, and the trajectory of clinical symptoms, including extrapyramidal rigidity, catatonic features, and mutism, may help clinicians suspect underlying cocaine-induced neurotoxicity. Toxic ingestion of drugs can cause neuronal inflammation and delayed post-hypoxic changes. Along with primary neurotoxicity from cocaine use, the patient may experience delayed hypoxic injury, other systemic manifestations like respiratory failure, cerebral ischemia, intravenous drug use-related endocarditis, complicating brain abscess, meningitis, encephalitis, and rarely myelopathy, as well as peripheral nervous system involvement causing polyradiculopathy. The majority of the cocaine-induced leukoencephalopathy is likely related to levamisole toxicity, which is an anthelmintic drug known to cause multifocal leukoencephalopathy, as 69% of the cocaine seized by the United States Drug Enforcement Agency showed the presence of levamisole mixed with cocaine. Patients with incomplete cognitive recovery after toxic substance use should trigger early neurological assessment with MRI brain, lumbar puncture, continuous video EEG, frequent neurochecks, and possible MR spectroscopy to narrow the differential diagnosis. Although there are anecdotal reports of vitamin C, vitamin E, and coenzyme Q use in similar cases, evidence lacks to support the use or show any remarkable benefit. There is a limited role for steroids, immunoglobulins, or plasma exchange in showing any improvement after toxic leukoencephalopathy. Despite aggressive care and early diagnosis, mortality, prolonged dependency on care, and cognitive impairment rates remain high with toxic leukoencephalopathy.
